# Genome-Wide Association Study of Salt Tolerance at the Seed Germination Stage in Flax (*Linum usitatissimum* L.)

**DOI:** 10.3390/genes13030486

**Published:** 2022-03-10

**Authors:** Xiao Li, Dongliang Guo, Min Xue, Gongze Li, Qingcheng Yan, Haixia Jiang, Huiqing Liu, Jiaxun Chen, Yanfang Gao, Lepeng Duan, Liqiong Xie

**Affiliations:** Xinjiang Key Laboratory of Biological Resources and Genetic Engineering, College of Life Science and Technology, Xinjiang University, Urumqi 830046, China; gaoxke@126.com (X.L.); gdl520jhx@163.com (D.G.); 17590102926@126.com (M.X.); ligongze816@outlook.com (G.L.); yqc18271692332@outlook.com (Q.Y.); jhx520gdl@163.com (H.J.); m_huiqing@163.com (H.L.); cjx18435138303@163.com (J.C.); zhizhen1505656805@163.com (Y.G.); duanlepeng1110@163.com (L.D.)

**Keywords:** GWAS, flax (*Linum usitatissimum* L.), salt tolerance, germination stage

## Abstract

Soil salinization seriously affects the growth and distribution of flax. However, there is little information about the salt tolerance of flax. In this study, the salt tolerance of 200 diverse flax accessions during the germination stage was evaluated, and then the Genome-wide Association Study (GWAS) was carried out based on the relative germination rate (RGR), relative shoot length (RSL) and relative root length (RRL), whereby quantitative trait loci (QTLs) related to salt tolerance were identified. The results showed that oil flax had a better salt tolerance than fiber flax. A total of 902 single nucleotide polymorphisms (SNPs) were identified on 15 chromosomes. These SNPs were integrated into 64 QTLs, explaining 14.48 to 29.38% (*R*^2^) of the phenotypic variation. In addition, 268 candidate genes were screened by combining previous transcriptome data and homologous gene annotation. Among them, *Lus10033213* is a single-point SNP repeat mapping gene, which encodes a Glutathione S-transferase (GST). This study is the first to use GWAS to excavate genes related to salt tolerance during the germination stage of flax. The results of this study provide important information for studying the genetic mechanism of salt tolerance of flax, and also provide the possibility to improve the salt tolerance of flax.

## 1. Introduction

Soil salinization is one of the main abiotic stress factors during crop growth and development, which causes poor crop growth, and leads to yield reduction or even death [1,2]. Under exposure to high concentrations of salt, ion toxicity destroyed the structure of the plasma membrane and ion imbalance promoted protein aggregation, then the physiological and metabolic activities of the plant were severely inhibited [3]. Nearly 300 million hectares of land in the world are affected by salt stress, and salinization or secondary salinization caused by water and soil pollution also increases significantly every year [4]. One of the most economical and effective ways to deal with soil salinization is to screen and cultivate salt tolerant crops [5]. The well-known sea (salt-tolerant) rice cultivation has been confirmed as improving the potential of saline-alkali land and ensuring food security [6]. Therefore, screening good salt-tolerant varieties and mining salt-tolerant genes are the effective approaches to dealing with environmental stress in recent years [7].

Although salt mainly affects the whole physiological process development in plants through ion toxicity and osmotic stress, it is reported that the stage of seed germination is more sensitive to changes in salt levels than other stages [8]. For example, in *Arabidopsis* [9], Sorghum [10], cowpea [11], etc. it is the stage most sensitive to salt during seed germination than at other stages of plant development. It is difficult for geminating seeds to absorb water and elongate roots in high-salt soil, and physiological drought caused by ion unbalance then affects the seedling planting [3]. Planting seeds that no longer sprout will limit crop yields [12]. Therefore, the germination stage deserves special attention when studying the salt tolerance of crops.

Flax (*Linum usitatissimum* L.) is a self-pollinating annual dicotyledonous crop, which can be divided into oil flax (Oil), fiber flax (Fiber) and oil-fiber dual purpose flax (OF) [13]. In contrast to the important crops of rice or wheat, flax is usually planted on barren land and even on saline alkali land in most of the flax countries, Canada, Russia and China, and the genetic diversity of the flax population under salt stress is still unclear [14,15,16]. Recently, a few studies were reported, all of which used transcriptome to study salt tolerance during the seedling stage of flax [17,18,19]. Yu et al. [17,18], by applying digital gene expression, investigated the transcriptome profile of flax under saline–alkaline stress. Wu et al. 2019 [19] also investigated the differentially expressed unigenes in flax under NaCl stress using RNA-Seq. No cases of salt tolerance during the gemination stage of flax were reported. Therefore, it is necessary to explore salt tolerance breeding resources for flax and identify elite resistant genes at the germination stage [14].

The Genome-wide Association Study (GWAS) is an effective strategy that uses single nucleotide polymorphisms (SNPs) as molecular genetic markers to find gene variations affecting complex traits [20]. With the advanced progress of gene sequencing and technical methods, GWAS has been successfully applied to determine plants’ salt tolerance genes [21], for example: *Arabidopsis* [22], rice [23,24,25,26], maize [27,28,29], soybean [30,31], cotton [32,33,34] etc. In flax, GWAS has been used to study agronomic traits [35], including pasmo resistance [36], seed yield and oil quality [37], seed size and weight [38], drought tolerance [39,40,41], imprinted genes [42] etc., but no research on salt tolerance was found.

In this study, we first evaluated the salt tolerance of 200 diverse flax accessions during the germination stage. Secondly, 674,074 SNPs were used for GWAS of salt tolerance related traits of flax during the germination stage, and the quantitative trait loci (QTLs) related to salt tolerance were identified. In combination with the previous transcriptome data, we screened some candidate genes in these QTLs. The purpose of this study is as follows: (a) to better understand the effect of salt stress during flax germination; (b) to identify SNPs related to salt tolerance and potential candidates during the germination stage by using GWAS.

## 2. Materials and Methods

### 2.1. Plant Materials

A GWAS population containing 200 diverse flax accessions was previously described [38].

### 2.2. Evaluation of Salt Tolerance and Phenotyping

The plump seeds of each accession were selected and placed in a 1.5 mL EP tube, soaked in 1% NaClO for 10 min, and then rinsed 2 to 3 times with distilled water. The 50 seeds were placed in a disposable plastic petri dish (Φ 90 mm) with two pieces of neutral filter paper (Φ 90 mm). Referring to our previous work (unpublished data), NaCl with concentrations of 250 mM (treatment group 1) and 100 mM (treatment group 2) were used for salt stress treatment. The 15 mL NaCl solution was added to the salt treatment group and 15 mL distilled water was added to the control group, which was then cultured in a constant temperature light incubator with 24 ± 2 °C, 16 h light/8 h dark and light intensity of 5000 lx. After 7 days of culture, the germination rate (GR) of seeds in each dish was calculated, and 10 flax plants were randomly selected to measure shoot length (SL) and root length (RL) using a scanner and Image J 1.8.0 software (https://imagej.nih.gov/ij/download.html, accessed on 4 March 2022). All flax accessions were laid out in a randomized complete-block design with three replications. We conducted three independent salt tolerance experiments in October (Environment 1, E1 for short), November (E2) and December (E3) 2019.

In the treatment group 1 (100 mM NaCl), we only measured shoot length (SL) and root length (RL); in the treatment group 2 (250 mM NaCl), we only measured the germination rate (GR). In order to eliminate the background differences among accessions, we used sterile water as the control group to measure the tolerance among varieties with sterile water as the control group. Phenotypic data were obtained as follows: relative germination rate (RGR), relative shoot length (RSL) and relative root length (RRL) (GR = number of germinated seeds/50) × 100%; relative value of each character under salt stress = measured value of treatment group/measured value of control group). In order to distinguish, three independent tests were designated RGR1, RSL1 and RRL1 (E1); RGR2, RSL2 and RRL2 (E2); RGR3, RSL3 and RRL3 (E3). The average value of the results of three independent tests was expressed as RGR-AVG, RSL-AVG and RRL-AVG. The weighted membership function (*D* value) was calculated by RGR-AVG, RSL-AVG and RRL-AVG to comprehensively evaluate the salt tolerance of flax germplasm at the germination stage. The calculation formula for *D* value is as follows:

(1) $$\mu (X_{i})\;=\;(X_{i}\;-\;X_{i\;\min})/\left(X_{i\;\max}\;-\;X_{i\;\min}\right)$$

(2) $$W_{i}\;=\;CV_{i}/\sum_{i=1}^{n}{CV}_{i}\;(i=1,\;2,\;3\cdots ,\;n)$$

(3) $$D\;=\;\sum_{i=1}^{n}[\mu \left(X_{i}\right){\cdot W}_{i}]\;(i=1,\;2,\;3\cdots ,\;n)$$

where, *X_i_* indicates the relative value of each material based on index *i*, *X_i_*_max_ and *X_i_* _min_ means maximum and minimum values of *X_i_*, *μ*(*X_i_*) means that each material is based on the membership function value of *X_i_*. The *CV_i_* is Coefficient of variation of *μ*(*X_i_*), *W_i_* means the ratio of the variation of *μ*(*X_i_*) to the total variation is the weight. The *D* value represents the sum of the product of the membership function value *μ*(*X_i_*) of each index and its weight *W_i_*, and represents multiple indexes to comprehensively evaluate the salt tolerance of each accession. 

### 2.3. Genome-Wide Association Study (GWAS)

Population structure, relative kinship and linkage disequilibrium (LD) analysis had already been analyzed in a previous study [38]. Here, a total of 674,074 SNPs (missing rate <0.2, coverage depth ≥3, MAF ≥0.05) were used for association analysis. A Genome-wide Association Study (GWAS) of salt tolerance traits in flax during germination stage was performed using general linear model (GLM) and mixed linear model (MLM) in Tassel 5.0 software [43]. The *p*-value thresholds were −log_10_(*P*) = 7.13 (GLM) and −log_10_(*P*) = 5.00 (MLM), as described in a previous study [38,44]. Results were visualized (Manhattan plots) using the R package “ggplot2” [45]. To integrate and determine the QTLs, we took the region within 54 Kb (the interval was considering the LD decay distances, *r*^2^ = 0.1; LD decay were drawn using an R scrip) (Supplementary Figure S1) around the significant SNP as a QTL (when two QTLs have overlapping regions, they are combined as the same QTL). A total of 286 preliminary QTLs were obtained. To improve the reliability of the results, we only retained 64 QTLs containing two or more distinct SNPs detected in different environments or different salt tolerance traits for further analysis. According to the GWAS associated loci, we constructed a LD Heatmap surrounding the Manhattan peak region, using the R package “LD Heatmap” [46].

### 2.4. Candidate Gene Prediction

We compared the QTLs located in this study with the previously reported QTLs related to the drought tolerance of flax. From the report of Soto-Cerda et al. 2019 [41], we selected the two QTLs that they located. From the report of Soto-Cerda et al. 2020 [39], we selected 15 favorite QTLs located by them. From the report of Sertse et al. 2021 [40], we selected 42 QTLs detected by more than two methods.

The 64 QTLs we screened contained 1591 flax genes. To further screen candidate genes, we compared our preliminary candidate genes with the transcriptome results of flax salt stress previously reported. From the report of Yu et al. 2014 [17], a total of 5331 differential expressed genes (DEGs) were obtained (Supplementary Table S1). From the report of Wu et al. 2019 [19], we only retained the differentially expressed genes that had gene ID, a total of 1634 DEGs (Supplementary Table S2). We kept these common genes as the candidate genes. We used *Arabidopsis* gene annotation and homologous gene function analysis to annotate all preliminary candidate genes (https://phytozome-next.jgi.doe.gov/, accessed on 4 March 2022). The genes associated with *Arabidopsis* salt stress were retained as candidate genes. Finally, we combined previous transcriptome data and gene annotation information to further screen candidate genes.

### 2.5. Haplotype Analysis

The haplotype analysis was carried out using Lead SNP in QTL. Differences between the haplotypes based on the lead SNP were statistically analyzed using *t*-tests.

### 2.6. Statistical Analysis

The phenotypic data were measured and recorded using the Microsoft Excel 2016 software. Statistical analysis of corresponding experimental data was carried out using GraphPad Prism 8 software and SPSS 26.0 software.

## 3. Results

### 3.1. Phenotypic Variation of Salt Tolerance in Seed Germination

**Figure 1:**
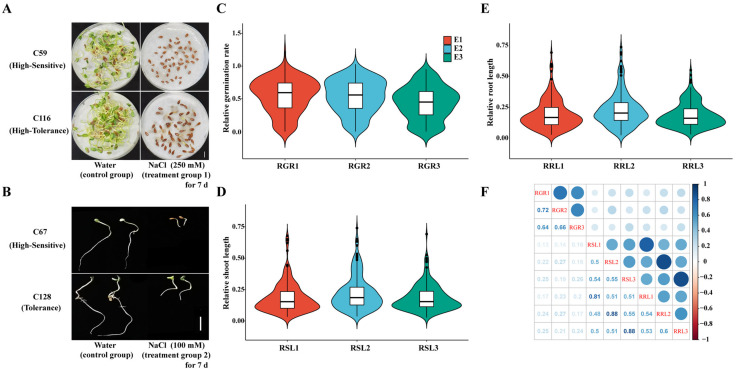
Phenotypic variation and correlation analysis of RGR, RSL and RRL traits. (**A**,**B**) Phenotypic diversity under salt stress during the germination stage; (**A**) Difference of GR under Salt Stress; (**B**) Difference of SL and RL under Salt Stress. Phenotype of flax accessions after 7 days of control and salt treatment. The photographs show representative seedlings. Scale bar, 1 cm; (**C**–**E**) Violin plot of phenotypic indexes; (**C**) RGR; (**D**) RSL; (**E**) RRL; (**F**) Correlation of all traits and environmental repeats. RGR1, RSL1 and RRL1 in environment 1 (E1). RGR2, RSL2 and RRL2 in environment 2 (E2). RGR3, RSL3 and RRL3 in environment 3 (E3).

The 200 flax accessions were tested under salt stress during the germination stage. The salt tolerance varied greatly among the flax accessions (Figure 1A,B). Compared with the control group, the salt treatment group significantly reduced germination rate (GR), shoot length (SL) and root length (RL) (*p* < 0.0001) (Supplementary Figure S2). It showed that salt stress in this concentration can differentiate well between all the tested accessions in the flax population. To normalize data from the different genetic backgrounds, the relative values were used to evaluate the salt tolerance of flax, which were: relative germination rate (RGR); relative shoot length (RSL); and relative root length (RRL). The phenotypic distribution showed continuous variation, and the variation of salt tolerance was rich (Figure 1C–E; Supplementary Table S3). The minimum value of RGR was 0.00 ~ the maximum value was 1.24 and the coefficient of variation (*CV*) was 47.36 to 57.97% in three environments; the range of RSL was 0.03~0.74 and the *CV* was 55.58 to 63.80%; the value of RRL ranged from 0.02 to 0.74, *CV* was 52.80 to 61.25% (in three environments) (Supplementary Table S4). Correlations were used to compare all traits with environmental repetitives (Figure 1F). Overall, the correlations between phenotypic data in different environments were all above 0.5, indicating that our screening was reliable. In the same environment, the correlation between RSL and RRL was above 0.8, indicating that the effects of salt stress on SL and RL were consistent.

### 3.2. Evaluation of Salt Tolerance of Flax Accessions

**Figure 2:**
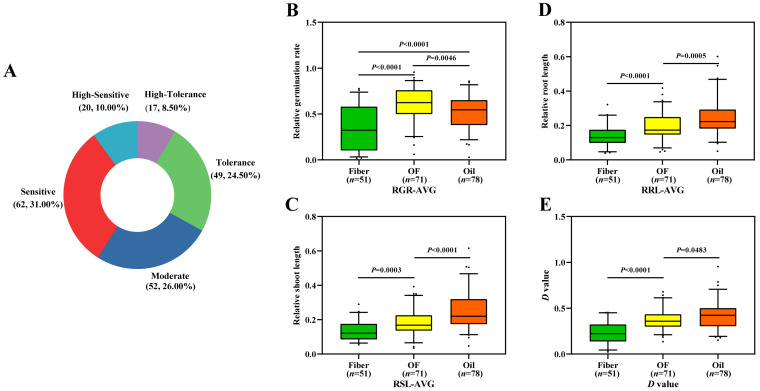
Evaluation of salt tolerance of flax germplasms. (**A**) Salt tolerance grades of 200 accessions of flax; (**B**–**D**) Distribution of salt tolerance indexes in subpopulations; (**B**) RGR-AVG; (**C**) RSL-AVG; (**D**) RRL-AVG; (**E**) *D* value. The difference between subpopulations was analyzed by *t*-tests.

The salt tolerance of 200 flax accessions was evaluated by the weighted membership function (*D* value) [47]. The *D* value of 200 flax accessions ranged from 0.02 to 0.95 (Supplementary Table S3). Among them, the *D* value of “ALSEE”, which originates from India, was highest at 0.95, indicating that it has the highest tolerance to salt stress; the *D* value of “Raisa”, which is from the Netherlands, was the smallest at 0.02, indicating that it was the most sensitive to salt stress. Sorting and cluster analysis was carried out according to the *D* value. The two-hundred flax accessions were divided into five groups, corresponding to five salt tolerance grades according to the *D* value (Supplementary Table S3). Among them, there were 17 high-tolerance grade accessions (8.50%), 49 tolerance grade accessions (24.50%), 52 moderate grade accessions (26.00%), 62 sensitive grade accessions (31.00%) and 20 high-sensitivity grade accessions (10.00%) (Figure 2A). To compare the difference of salt tolerance among different flax subpopulations in the germination stage, we analyzed the difference of various salt tolerance indices in the subpopulations (Figure 2B–E; Supplementary Figure S3). The results showed that the salt tolerance indices (RGR, RSL, RRL and *D* values) of the oil flax subpopulation were significantly higher than those of the OF flax and fiber flax. These results showed that the oil flax subpopulation had a stronger tolerance to salt stress than the OF flax and fiber flax, and the fiber flax had the weaker tolerance to salt stress. 

### 3.3. Genome-Wide Association Study

**Figure 3:**
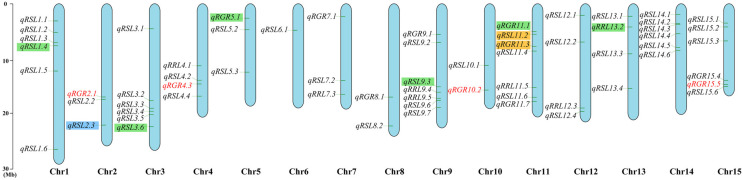
Physical map of 64 QTLs. QTLs repeatedly detected at the same SNP locus in three environments are marked in red. QTLs overlapping with those of Soto-Cerda et al. 2019 were marked in orange boxes. QTLs overlapping with those of Sertse et al. 2021 were marked in green boxes. QTLs overlapping with those of Soto-Cerda et al. 2020 and Sertse et al. 2021 were marked in blue boxes.

To gain insight into genetic variations associated with salt tolerance during the germination stage of flax, a total of 674,074 SNPs were used for GWAS analysis of salt tolerance traits. Using GLM and MLM models, we identified 1075 SNPs (after deduplication, there are a total of 902 SNPs) significantly related to salt-related RGR, RSL and RRL (Supplementary Figures S4–S6; Supplementary Table S5). These SNPs are located on all 15 chromosomes. Among 1075 SNPs, 709 SNPs were identified in GLM and 366 SNPs in MLM. These SNPs were integrated into 64 QTLs, which explained the 14.48% to 29.38% (*R*^2^) phenotypic variation (Figure 3 and Supplementary Table S6). Among these 64 QTLs, 10 were detected in all three environments (Table 1). SNPs contained in *qRGR2.1*, *qRGR4.3*, *qRGR10.2*, and *qRGR15.5* were repeatedly detected in three environments (Figure 3, Table 1). 

**Figure 4:**
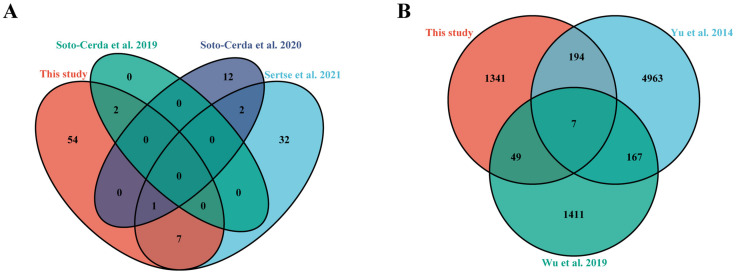
Compared with previous reports. (**A**) Comparison of the 64 QTLs involved in flax salt-tolerance detected in this study and the QTLs regulating drought tolerance reported previously; (**B**) Comparison of the 1591 preliminary candidate genes involved in flax salt-tolerance detected in this study and the transcriptome genes with salt stress reported previously.

Because no definite QTLs related to salt tolerance have been reported in flax and it had been confirmed that drought stress shares some signal and metabolism pathways in many plants [48], we compared the QTLs related to drought stress reported by previous studies (Figure 4A). The results showed that the 64 QTLs in our study overlapped with the two sites in Soto-Cerda et al. 2019 [41], which were all located on chromosome 11; there was 1 QTL located on chromosome 2 that overlapped with the results of Soto-Cerda et al. 2020 [39]; there were 8 QTLs that overlapped with Sertse et al. 2021 [40] (2 QTLs overlapped with *qRGR11.1*), which were located on chromosomes 1, 2, 3, 5, 9, 11 and 13, respectively. A common overlapping QTL (*qRSL2.3*) in these studies was found on chromosome 2 (Figure 3). Our results indicated that these QTLs are involved in the response not only to drought stress, but also to salt stress.

### 3.4. Screening Candidate Genes

The 64 QTLs contained a total of 1591 flax genes (Supplementary Table S7). To further screen the candidate genes, comparison of the 1591 preliminary candidate genes was made in this study and the transcriptome genes with salt stress reported previously. The results showed that there were 201 overlapping genes between the 1591 preliminary candidate genes and Yu et al.’s DEGs data, 2014 [17], of which 91 were upregulated and 110 were downregulated; there were 56 overlapping genes between the 1591 preliminary candidate genes and Wu at al.’s RNA-seq data 2019 [19], of which 38 are upregulated and 18 are downregulated (Figure 4B). Among them, seven common genes overlapped with Yu et al., 2014, and Wu et al., 2019, which includes five genes that were predicted to be related to salt tolerance based on their function in other plants, and there were two genes (*Lus10041550* and *Lus10013312*) without functional annotation (Table 2) [49,50,51,52,53]. Finally, combining previous transcriptome data and gene annotation information, we further screened a total of 268 candidate genes potentially related to salt tolerance in seed germination stage (Supplementary Table S8).

**Figure 5:**
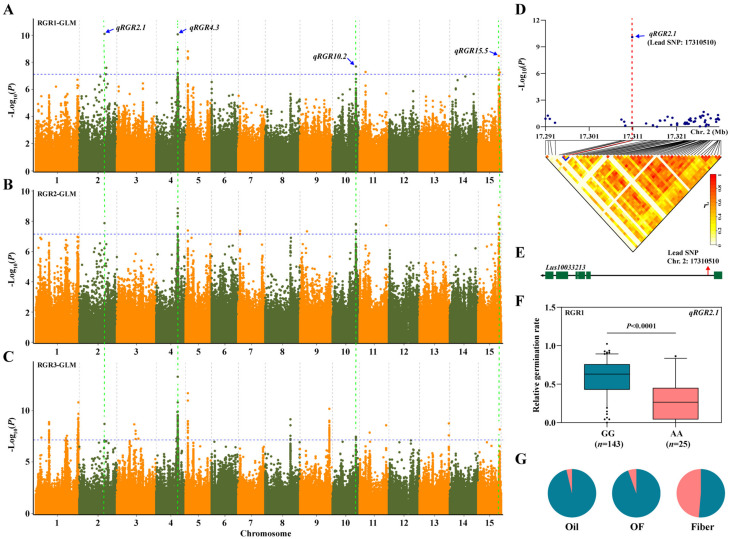
GWAS for RGR traits, and candidate genes were obtained for the peak region on chromosome 2. (**A**–**C**) Manhattan plots based on GLM; (**A**) RGR1; (**B**) RGR2; (**C**) RGR3. The horizontal blue dotted line indicates the threshold, and the points above the threshold are significant SNPs. The point indicated by the blue arrow is the lead SNP in the repeatedly detected QTLs; (**D**) Local Manhattan plot (top) and LD heatmap (bottom) surrounding the peak on chromosome 2. The blue arrow indicates a significant SNP (Lead SNP: 17310510) in *qRGR2.3;* (**E**) Gene structure of *Lus10033213;* (**F**) Boxplot for haplotypes based on the lead SNP in *qRGR2.3;* (**G**) The distribution of allele frequencies of strong SNP are located in *qRGR2.3* which was distributed in Oil, OF and Fiber subpopulations. The GG and AA alleles are shown in blue and pink, respectively. The difference between haplotypes was analyzed by *t* tests.

In addition, we noticed that there was a SNP (Position: Chr2-17310510) that was repeatedly detected by three environments on chromosome 2, which was located in *qRGR2.1* (Figure 5A–C). A local Manhattan plot (top) showed that this is a single significant SNP. The LD heatmap (bottom) also shows that this SNP is not linked with other sites (Figure 5D). This SNP is located at *Lus10033123* (Figure 5E), which encodes a Glutathione S-transferase (GST). It has been reported that overexpression of GST can improve salt tolerance in *Arabidopsis* [54]. Haplotype analysis of the allele based on this SNP showed that flax accessions containing the GG haplotype were more tolerant to salt than those in the AA haplotype (Figure 5F). Meanwhile, we found that leader SNPs displayed a subpopulation specific haplotype in repeated QTLs (*qRGR2.1*, *qRGR4.3*, *qRGR10.2*, *qRGR15.5*), which were screened by GWAS. Compared with the fiber subpopulation, there are more favorable alleles related to salt tolerance in the oil subpopulation. (Figure 5G and Supplementary Figure S7). This result was consistent with the phenotypic indices difference in different flax subpopulations (Figure 2B–E; Supplementary Figure S3). It again shows that oil flax has a higher salt tolerance than fiber flax. 

## 4. Discussion

Seed germination rate, shoot length, and root length are commonly used as indicators to assess the salt tolerance of plant germination [55]. In our previous study, it was found that when flax seeds were treated with 100 mM NaCl stress, the variation in SL and RL was the largest, while the change of GR was not significant. Until 250 mM NaCl treatment, GR can be used to distinguish different varieties in the population (data unpublished). Our phenotypic results also verified the previous results (Figure 1C–E). This suggests that the response thresholds of various indicators to salt stress are different. Therefore, in this study, we used these two NaCl concentration treatment groups. Although RGR can well distinguish the salt tolerance of different flax germplasm, it showed a low correlation with RSL or RRL (Figure 1D). This phenomenon has also been reported in some crops (rice [56], chicory [57], sesame [58], etc.), and different parts of crops have a different sensitivity to salt stress. It is generally believed that the damage of high salt to plants is mainly caused by osmotic stress and ion toxicity [59]. The low water potential formed by high ion concentration in the external environment inhibits the imbibition of seeds, thus inhibiting seed germination [60], while the growth of seedling radicles and embryos is affected by the synergistic effect of osmotic stress and ion toxicity.

Although the tolerance phenotype showed continuous variation, the variation in different subgroups in our tested flax population was obvious. The RGR, RSL, RRL and *D* values of the oil flax subpopulation were significantly higher than those of the fiber flax subpopulation (Figure 2B–E; Supplementary Figure S3). Compared with the fiber flax subpopulation, the oil flax subpopulation contained more favorable alleles related to salt tolerance (Figure 5F–G, Supplementary Figure S7). Previous studies believed that oil flax is the ancestor of cultivated flax, fiber flax is domesticated from oil flax, and oil and fiber flax are the intermediate types in the domestication process from oil to fiber flax [38,61,62]. In this paper, our results confirmed that oil flax has a richer genetic diversity faced with salt stress. Therefore, oil flax may contain more resistance sites related to abiotic stress, and the screening of resistant germplasm and resistance genes should be focused more on oil flax.

To the best of our knowledge, this study is the first to use GWAS to excavate genes related to salt tolerance during the germination stage of flax. There were 902 significant SNPs which were located in 64 QTLs, which were detected using GWAS of salt-tolerance related traits of 200 diverse flax accessions. Due to plants possessing interconnected regulatory pathways to respond to kinds of environmental stress, it is generally believed that plants share several common mechanisms to cope with drought and salt stress [44]. Since there are no definite reported QTLs related to salt tolerance of flax at present, we compared the QTLs related to drought stress in previous studies (Figure 4B). We noticed that some of the QTLs we identified overlapped with the QTLs of flax response to drought stress reported. Among them, the *qRSL2.3* localized on chromosome 2 was co-located with the results of the previous two studies, in which it contained a gene (*Lus10013205*) annotated as *NAC* gene. Expression of *NAC* has been reported to improve both salt and drought tolerance in *Arabidopsis* [63], wheat [64], and tomato [65]. This indicates that flax may have common response genes in the face of different abiotic stresses.

In this study, a QTL contains more than 20 genes on average. Therefore, it is difficult to identify the key candidate genes in these QTLs. For non-model organisms, it is a feasible and effective method to screen candidate genes by combining the transcriptome data and the homology annotation [66]. In this study, followed this strategy, two transcriptome profilings related to salt tolerance of flax were integrated and 268 candidate genes were screened. Among these candidate genes, a large number of transcription factors were found, including *NAC* (e.g.: *Lus10041534*, *Lus10004531*); *MYB* (e.g.: *Lus10034372*, *Lus10034372*); *WRKY* (e.g.: *Lus10018815*, *Lus10007907*); *bZIP* (e.g.: *Lus10034296*, *Lus10029101*); *ERF* (e.g.: *Lus10022935*, *Lus10029331*); and *AP2* (e.g.: *Lus10007192*, *Lus10037448*). Previous studies have shown that the homologous genes of these transcription factors in other plants mainly play an important role in the early sensing mechanism of salt stress by further regulating the expression of various downstream genes [67,68,69].

In addition, the candidate genes of our study also include genes whose homology has been functionally verified under salt stress in other plants, mainly including *GST* (e.g.: *Lus10033213*, *Lus10007897*); *ABC* (e.g.: *Lus10034387*, *Lus10037947*); *RING* (e.g.: *Lus10027744*, *Lus10018817*); *C2H2* (e.g.: *Lus10039246*, *Lus10037942*); *SnRK2* (e.g.: *Lus10004748*) etc., these genes are verified as being involved in many biological processes. Overexpression of *GST* can reduce the content of MDA under salt stress, so as to improve the salt tolerance in *Arabidopsis* [54]. The ABC transporter genes are known to play an important role in the salt tolerance mechanism in rice by acting as transmembrane transporters [70,71]. The RING finger ubiquitin E3 ligase SDIR1 targets SDIR1-INTERACTING PROTEIN1 for degradation to modulate the salt stress response and ABA signaling in *Arabidopsis* [49]. Ascorbic acid (AsA) promotes stress tolerance by scavenging reactive oxygen species (ROS). The C2H2 zinc-finger protein SlZF3 regulates AsA synthesis and salt tolerance by interacting with CSN5B [72]. Overexpression of *NtSnRK2.2* enhances salt tolerance in *Nicotiana tabacum* by regulating carbohydrate metabolism and lateral root development [73]. This suggests that the set of candidate genes we assessed may be involved in the regulation of salt tolerance in flax germination in multiple ways. This also shows that our method of identifying salt tolerance genes is reliable. However, the specific role of these genes in flax needs further verification. In addition, we also found multiple duplicate genes in the same QTL, for example, *Lus10014812* and *Lus10014810*. The discovery of these salt tolerant repetitive genes may imply that the evolution of repetitive genes can make flax better adapt to environmental changes under the action of gene mutation and natural selection.

Although the genes related to salt tolerance at the germination stage of flax were excavated in this study, the tolerance of crops to salt stress was different at different development stages [74]. We also need to study the salt tolerance of flax in other development stages and the whole growth period. Our study provides lays an important foundation for the genetic improvement of salt tolerance in flax in the future.

## 5. Conclusions

In this study, we evaluated salt tolerance of 200 flax accessions at germination stage and screened salt tolerant germplasms. The 902 significant SNPs were detected from the GWAS of salt tolerance related traits of 200 diverse flax accessions during the germination stage. These SNPs were integrated into 64 QTLs, explaining 14.48 to 29.38% (*R*^2^) of the phenotypic variation. The 268 candidate genes were screened by combining transcriptome and homologous gene annotation. Among them, *Lus10033213* is a single-point SNP repeat mapping gene, which encodes a Glutathione S-transferase (GST). Oil flax has a higher salt tolerance than fiber flax at germination stages. Our study provides a basis to understand the genetic basis of plant salt tolerance and lays an important foundation for the genetic improvement of salt tolerance in flax in the future.

## Figures and Tables

**Table 1:** Ten QTLs were detected in all three environments.

QTL ^1^	Traits	Lead SNP ^2^	Model	Chr	Position (bp) ^3^	−log_10_ (*P*)	*R*^2^ (%) ^4^
*qRGR2.1*	RGR1 *, RGR2, RGR3	SNP92745	GLM *, MLM	2	17,310,510 ^#^	10.09	24.53
*qRGR4.3*	RGR1, RGR2, RGR3 *, RSL1	SNP192459	GLM *, MLM	4	14,692,534 ^#^	13.27	29.38
*qRSL4.4*	RSL2, RSL3 *, RRL1	SNP194815	GLM *, MLM	4	17,154,148	7.24	15.41
*qRGR5.1*	RGR1 *, RGR2, RGR3	SNP206061	GLM *	5	1,440,001	8.80	21.88
*qRSL6.1*	RSL1 *, RSL2, RSL3	SNP253553	GLM *, MLM	6	3,866,787	7.87	17.01
*qRGR10.2*	RGR1 *, RGR2, RGR3, RSL3	SNP439280	GLM *, MLM	10	15,921,726 ^#^	7.69	19.45
*qRRL11.5*	RSL2, RSL3, RRL1 *, RRL2	SNP470912	GLM *, MLM	11	15,408,746	9.09	20.39
*qRSL14.5*	RSL1 *, RSL2, RSL3	SNP610085	GLM *, MLM	14	7,354,242	10.68	23.43
*qRSL14.6*	RSL1, RSL2, RSL3 *, RRL2	SNP610721	GLM, MLM *	14	7,956,601	6.45	18.99
*qRGR15.5*	RGR1, RGR2, RGR3 *, RSL1, RSL3, RRL3	SNP672146	GLM *, MLM	15	14,770,681 ^#^	8.16	19.73

^1^ QTL = quantitative trait locus. Naming method: “q” + “target trait” + “chromosome number” + “.” + “order number”; ^2^ Lead SNP = The most significant SNP in QTL; ^3^ Position (bp) is the Lead SNP; ^4^ *R*^2^ (%) = Coefficient of determination indicating percent phenotypic variance explained. * Connected to Lead SNPs. ^#^ Indicates this position is repeatedly detected in three environments.

**Table 2:** Seven genes consistent with the transcriptome genes with salt stress reported previously.

Gene	QTL	Chr	*Arabidopsis* Gene	*Arabidopsis* Functional Annotation
*Lus10012628*	*qRSL3.6*	3	*AT5G53110.1*	RING/U-box superfamily protein [49]
*Lus10041550*	*qRGR4.3*	4	*AT3G27030.1*	Unknown protein
*Lus10026381*	*qRGR11.1*	11	*AT1G07160.1*	Protein phosphatase 2C family protein [50]
*Lus10026376*	*qRGR11.1*	11	*AT3G47340.1*	Glutamine-dependent asparagine synthase 1 [51]
*Lus10006732*	*qRSL12.1*	12	*AT4G11170.1*	Disease resistance protein (TIR-NBS-LRR class) family [52]
*Lus10013312*	*qRSL14.3*	14	*AT3G11590.1*	Unknown protein
*Lus10037940*	*qRGR15.4*	15	*AT5G03530.1*	RAB GTPase homolog C2A [53]

## Data Availability

All sequencing data generated for this study can be found in the NCBI using accession number PRJNA590636 (https://www.ncbi.nlm.nih.gov/bioproject/PRJNA590636, accessed on 4 March 2022).

## Supplementary Materials

The following supporting information can be downloaded at: https://www.mdpi.com/article/10.3390/genes13030486/s1, Figure S1: Linkage disequilibrium (LD) decay distance; Figure S2: Comparison of GR, SL, RL under control (CK) and treatment; Figure S3: Distribution of salt tolerance indexes in subpopulations; Figure S4: Genome-wide association study (GWAS) for RGR; Figure S5: Genome-wide association study (GWAS) for RSL; Figure S6: Genome-wide association study (GWAS) for RRL; Figure S7: Boxplots for haplotypes at the lead SNP in QTLs and allele frequency differences among Oil, OF and Fiber subpopulations; Table S1: The DEGs of Yu et al. 2014; Table S2: The DEGs of Wu et al. 2019; Table S3: The two-hundred accessions of flax related information, stress indices across three environments and salt tolerance grade; Table S4: Descriptive statistics of salt tolerance indexs; Table S5: Significant SNPs obtained by GWAS; Table S6: The sixty-four QTLs obtained from significant SNPs integration; Table S7: Flax genes contained in 64 QTLs; Table S8: Candidate genes.
