# Supplementary material for: Genome-Wide Association Study of Salt Tolerance at the Seed Germination Stage in Flax (Linum usitatissimum L.)
Source: Genes (Basel). 2022 Mar 10;13(3):486. doi: 10.3390/genes13030486 (PMC8949523; doi:10.3390/genes13030486)
Supplement: Supplementary file 1 [file genes-13-00486-s001.zip › Supplementary files/Supplementary Figures.pdf]

Supplementary Figures

Genome-Wide Association Study of Salt Tolerance at the Seed Germination Stage in Flax (*Linum usitatissimum* L.)

Xiao Li, Dongliang Guo, Min Xue, Gongze Li, Qingcheng Yan, Haixia Jiang, Huiqing Liu, Jiaxun Chen, Yanfang Gao, Lepeng Duan and Liqiong Xie*

[1] Xinjiang Key Laboratory of Biological Resources and Genetic Engineering, College of Life Science and Technology, Xinjiang University, Urumqi 830046, Xinjiang, China; gaoxke@126.com

* Correspondence: picea@xju.edu.cn

Supplementary Figure S1. Linkage disequilibrium (LD) decay distance.

Supplementary Figure S2. Comparison of GR, SL, RL under control (CK) and treatment.

Supplementary Figure S3. Distribution of salt tolerance indexes in subpopulations.

Supplementary Figure S4. Genome-wide association study (GWAS) for RGR.

Supplementary Figure S5. Genome-wide association study (GWAS) for RSL.

Supplementary Figure S6. Genome-wide association study (GWAS) for RRL.

Supplementary Figure S7. Boxplots for haplotypes at the lead SNP in QTLs and allele frequency differences among Oil, OF and Fiber subpopulations.

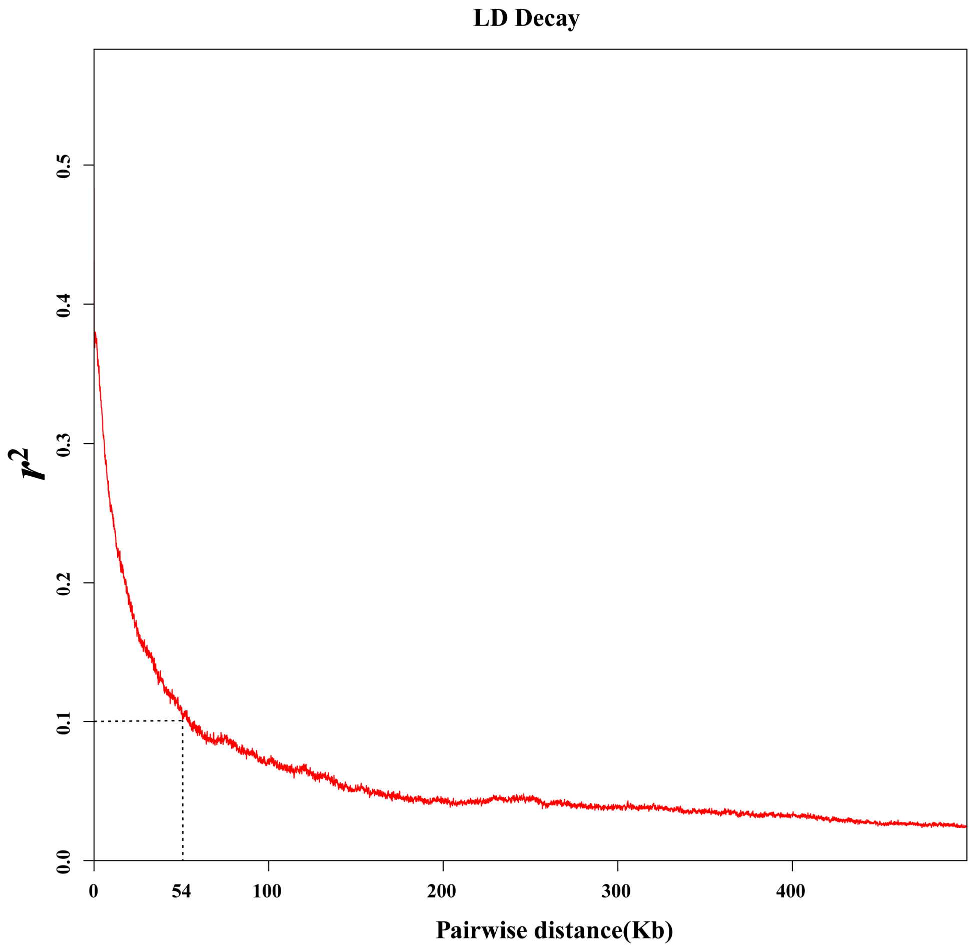

Supplementary Figure S1. Linkage disequilibrium (LD) decay distance.

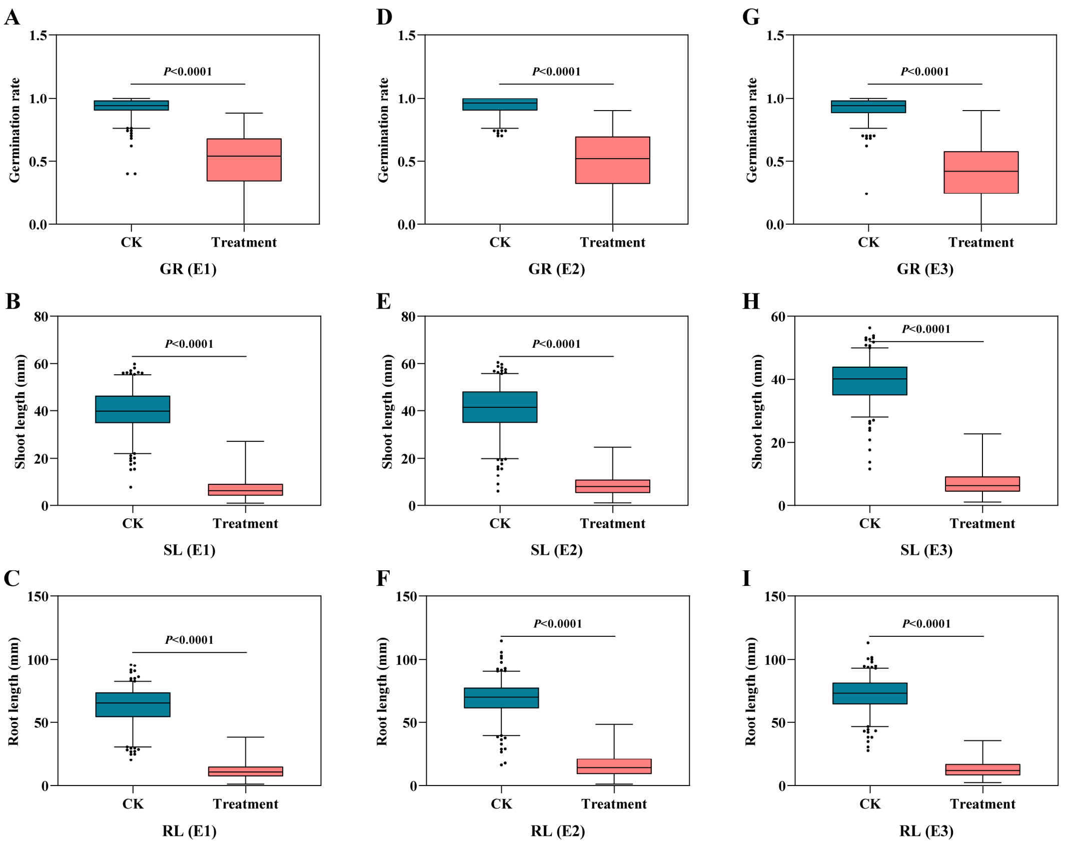

Supplementary Figure S2. Comparison of GR, SL, RL under control (CK) and treatment. (**A**–**C**) In environment 1 (E1); (**D**–**F**) In environment 2 (E2); (**G**–**I**) In environment 3 (E3). The difference between subpopulations was analyzed by *t* tests.

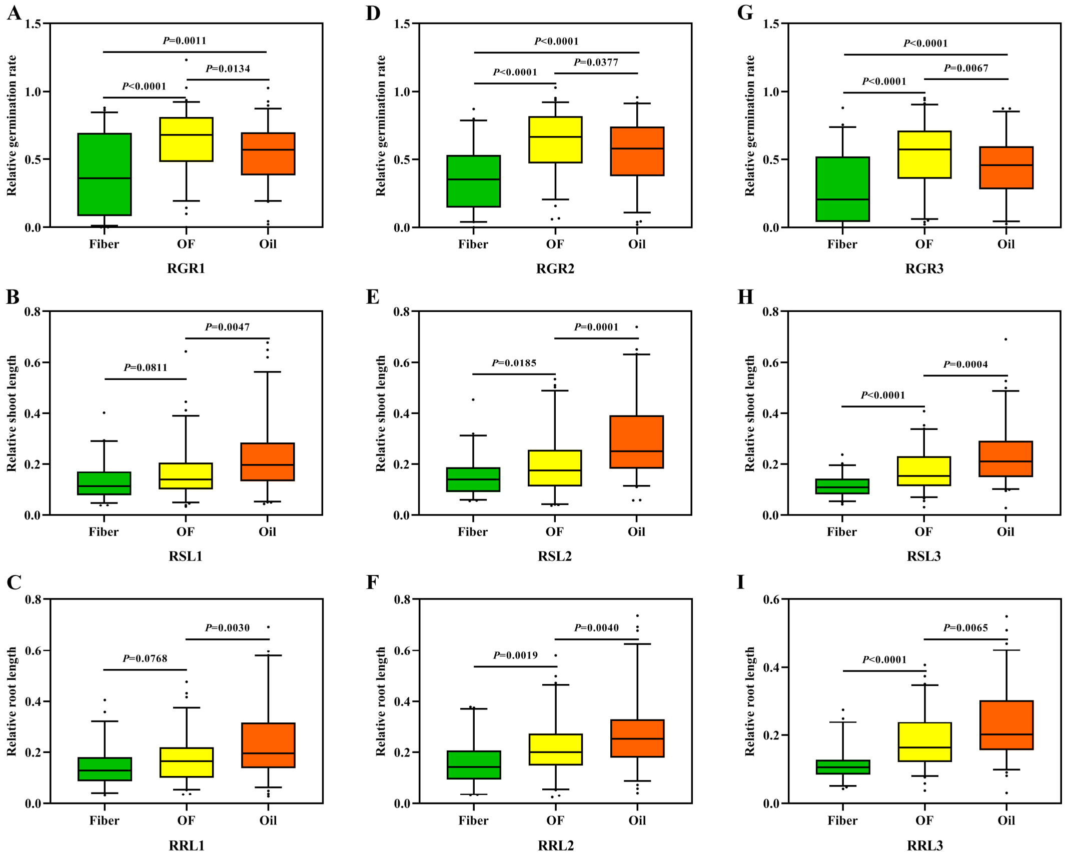

Supplementary Figure S3. Distribution of salt tolerance indexes in subpopulations. (**A**–**C**) In environment 1 (E1); (**D**–**F**) In environment 2 (E2); (**G**–**I**) In environment 3 (E3). The difference between subpopulations was analyzed by *t* tests.

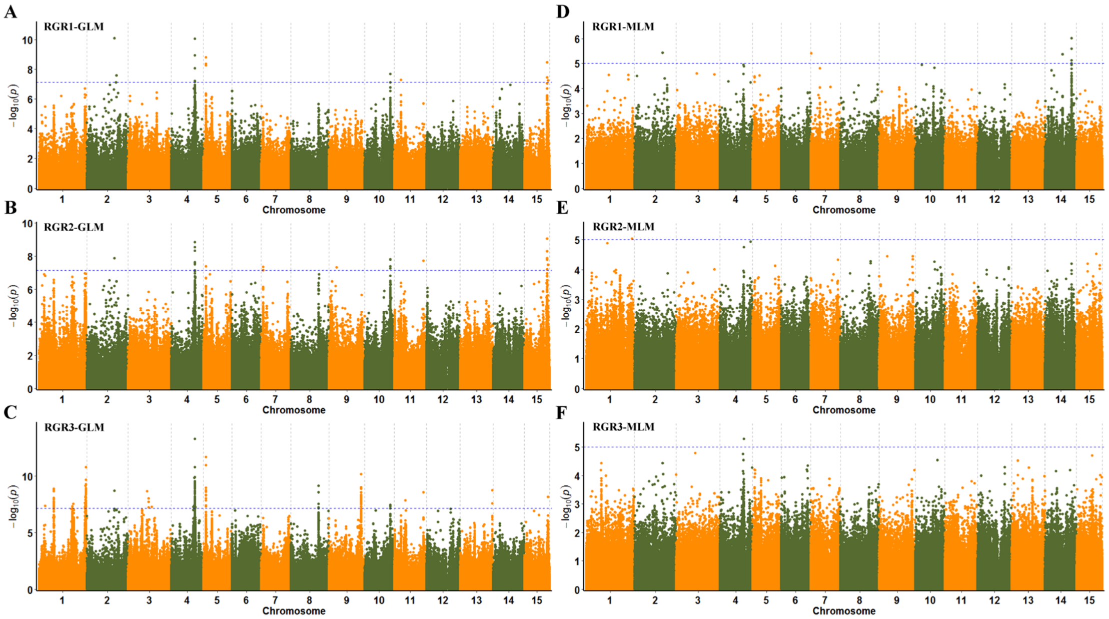

Supplementary Figure S4. Genome-wide association study (GWAS) for RGR. (**A**–**C**) Manhattan plots based on GLM; (**D**–**F**) Manhattan plots based on MLM; (**A**, **D**) RGR1; (**B**, **E**) RGR2; (**C**, **F**) RGR3. The blue dotted line indicates the threshold, and the points above the threshold are significant SNPs.

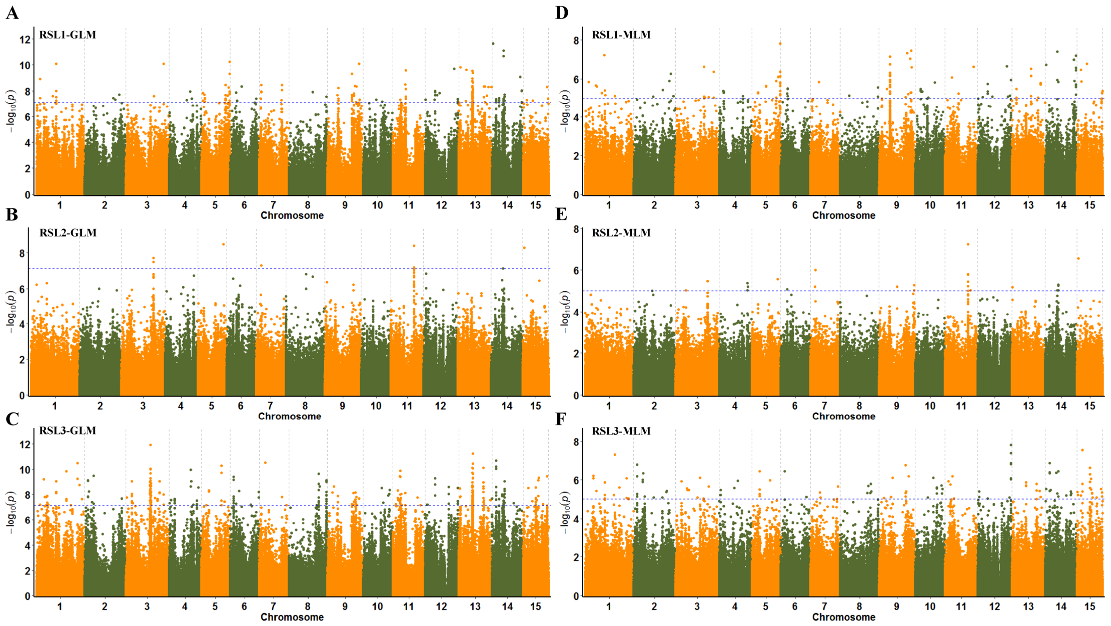

Supplementary Figure S5. Genome-wide association study (GWAS) for RSL. (**A**–**C**) Manhattan plots based on GLM; (**D**–**F**) Manhattan plots based on MLM; (**A**, **D**) RSL1. (**B**, **E**) RSL2; (**C**, **F**) RSL3. The blue dotted line indicates the threshold, and the points above the threshold are significant SNPs.

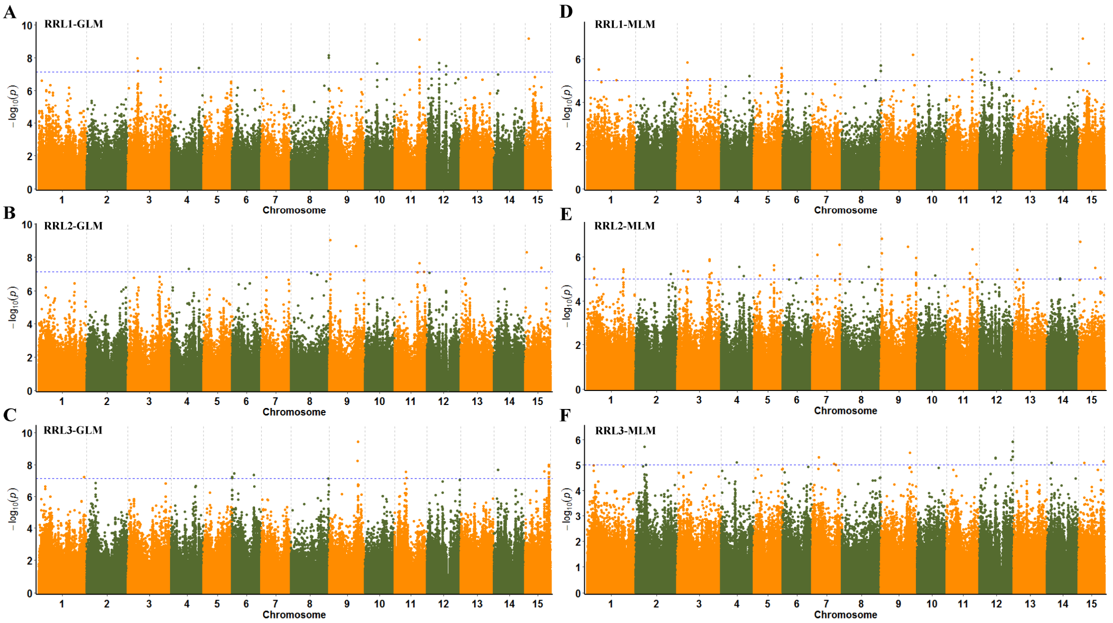

Supplementary Figure S6. Genome-wide association study (GWAS) for RRL. (**A**–**C**) Manhattan plots based on GLM; (**D**–**F**) Manhattan plots based on MLM; (**A**, **D**) RRL1. (**B**, **E**) RRL2; (**C**, **F**) RRL3. The blue dotted line indicates the threshold, and the points above the threshold are significant SNPs.

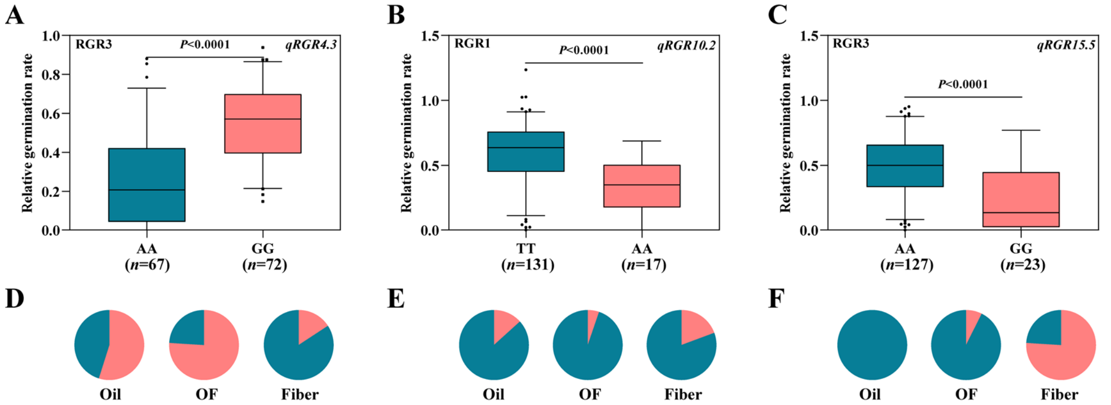

Supplementary Figure S7. Boxplots for haplotypes at the lead SNP in QTLs and allele frequency differences among Oil, OF and Fiber subpopulations. (**A**–**C**) Boxplots for haplotypes at the lead SNP in QTLs; (**A**) *qRGR4.3*; (**B**) *qRGR10.2*; (**C**) *qRGR15.5*; (**D**–**F**) The distribution of allele frequencies of strong SNPs are located in QTLs were distributed in Oil, OF and Fiber subpopulations; (**D**) *qRGR4.3*. The AA and GG alleles are shown in blue and pink, respectively; (**E**) *qRGR10.2*. The TT and AA alleles are shown in blue and pink, respectively; (**F**) *qRGR15.5*. The AA and GG alleles are shown in blue and pink, respectively. The difference between haplotypes was analyzed by *t* tests.
